# A Novel Role for miR‐1305 in Regulation of Pluripotency‐Differentiation Balance, Cell Cycle, and Apoptosis in Human Pluripotent Stem Cells

**DOI:** 10.1002/stem.2444

**Published:** 2016-07-11

**Authors:** Shibo Jin, Joseph Collin, Lili Zhu, David Montaner, Lyle Armstrong, Irina Neganova, Majlinda Lako

**Affiliations:** ^1^ Institute of Genetic MedicineNewcastle UniversityUK; ^2^ Centro De Investigacion Principe FelipeValenciaSpain

## Abstract

Human embryonic stem cells (hESCs) and human induced pluripotent stem cells (hiPSCs) are defined as pluripotent in view of their self‐renewal ability and potential to differentiate to cells of all three germ layers. Recent studies have indicated that microRNAs (miRNAs) play an important role in the maintenance of pluripotency and cell cycle regulation. We used a microarray based approach to identify miRNAs that were enriched in hESCs when compared to differentiated cells and at the same time showed significant expression changes between different phases of cell cycle. We identified 34 candidate miRNAs and performed functional studies on one of these, miR‐1305, which showed the highest expression change during cell cycle transition. Overexpression of miR‐1305 induced differentiation of pluripotent stem cells, increased cell apoptosis and sped up G1/S transition, while its downregulation facilitated the maintenance of pluripotency and increased cell survival. Using target prediction software and luciferase based reporter assays we identified *POLR3G* as a downstream target by which miR‐1305 regulates the fine balance between maintenance of pluripotency and onset of differentiation. Overexpression of *POLR3G* rescued pluripotent stem cell differentiation induced by miR‐1305 overexpression. In contrast, knock‐down of *POLR3G* expression abolished the miR‐1305‐knockdown mediated enhancement of pluripotency, thus validating its role as miR‐1305 target in human pluripotent stem cells. Together our data point to an important role for miR‐1305 as a novel regulator of pluripotency, cell survival and cell cycle and uncovers new mechanisms and networks by which these processes are intertwined in human pluripotent stem cells. Stem Cells *2016;34:2306–2317*

**Significance Statement**

Pluripotent stem cells hold great promise for basic biology studies, understanding of human development and for cell based replacement therapies of inherited and age related degenerative diseases. However, to fully realise their potential and to avoid safety concerns, directed, and efficient strategies must be developed to control their differentiation along desired lineages. This requires a fundamental understanding of the molecular mechanisms underlying self‐renewal and maintenance of pluripotency. Along with signalling pathways, transcription factors, and epigenetic regulators, miRNAs are emerging as important regulators in the maintenance of pluripotency and the cell cycle. In this present study, we have identified a novel regulator of pluripotency, cell survival and cell cycle, namely miR‐1305 and have provided evidence that miR‐1305 regulates the pluripotency‐differentiation balance by directly binding to the 3′UTR of POLR3G pluripotency factor and regulating its expression.

## Introduction

Human pluripotent stem cells have two unique properties: unlimited self‐renewal and pluripotency 1, 2. MicroRNAs (miRNAs) are a class of small (18–25 nucleotides) regulatory non‐coding RNAs that are thought to regulate gene expression through sequence‐specific base pairing with target mRNAs 3. Recent studies have highlighted an important role for miRNAs in maintaining the delicate balance between pluripotency and the onset of differentiation through close links with the pluripotency transcription network. Key pluripotency factors (Sox2, Oct4, and Nanog) can regulate the expression of pluripotent stem cell (ESC) specific miRNAs (for example mir‐290 and 302 clusters) by binding to their promoters 4, 5. In turn, miRNAs (for example miR‐134, miR‐296, and miR‐470) can target the coding sequence of *Sox2*, *Oct4*, and *Nanog* in various combinations leading to the loss of pluripotency and onset of differentiation 6, 7. Furthermore, miRNAs (mir‐302, 367, 145, etc) have been implicated in somatic cell induced reprogramming through regulating the expression of master pluripotency factors, epigenetic factors and genes involved in mesenchymal to epithelial transition 8, 9.

Rapid cell cycle progression is a distinct feature of pluripotent stem cells. A short G1 phase has been considered important for the maintenance of pluripotency by limiting the window of opportunity during which pluripotent stem cells are exposed to differentiation cues 10, 11, 12. Recent evidence suggests that miRNAs regulate many genes that are involved in cell cycle progression in ESCs 13. Depletion of miRNAs through knockdown of *Dicer* and *Drosha* in murine ESC results in slower proliferation and accumulation of cells in G1 phase of the cell cycle 14, 15 which can be rescued by overexpression of the mir‐290/302 cluster 16 and early differentiation factors (*Wee1* and *Fbx15*) 16, 17.

Knockdown of *DICER* or *DROSHA* in human ESCs (hESCs) also results in reduced generation of miRNAs and accumulation of cells in the G1 and G2/M phases of cell cycle 18. The G1 blockage can be rescued by overexpression of miR‐372 which has been shown to regulate the cyclin E/Cdk2 pathway in G1/S transition by inhibiting the cell cycle inhibitor CDKN1A (p21) 18. The G2/M cell accumulation can be reversed by the overexpression of miR‐195 which regulates *WEE1* kinase, a known inhibitor of cyclin B/Cdk1 which is necessary for G2/M transition 18. Moreover, the miR‐302 cluster, which is the most enriched miRNA cluster in hESCs and important for the maintenance of pluripotency also promotes G1/S transition by inhibiting cyclin D1. In support of this role, it has been shown that inhibition of miR‐302 induces cell accumulation in G1 phase and the onset of differentiation 5, 19. Together these published data indicate that ESCs specific miRNAs have a central role in expediting the G1‐S transition and promoting cellular proliferation.

In this present study, we have identified a novel regulator of early differentiation events, cell survival and cell cycle, namely miR‐1305 and have provided evidence that miR‐1305 regulates the pluripotency‐differentiation balance by directly binding to the 3′UTR of *POLR3G* pluripotency factor and regulating its expression.

## Results

### Microarray‐Based Expression Profiling at Different Stages of the hESCs Cell Cycle and Differentiation Process

Our previous studies have shown that cell cycle regulation and pluripotency are two critically intertwined processes that may be regulated by key pluripotency factors including miRNAs 20, 21 . To identify miRNA candidates which are likely to regulate the pluripotent phenotype as well as the cell cycle, we synchronised hESCs at different cell cycle stages (G1, S, and G2/M; Supporting Information Fig. 1A). RNAs obtained from these samples as well as human placental fibroblasts and unsynchronised hESCs were used for miRNA screening analysis using the Agilent human miRNA (V3) 8X15K microarray (Agilent, G4470) containing 866 human and 89 human viral miRNAs probes.

**Figure 1:**
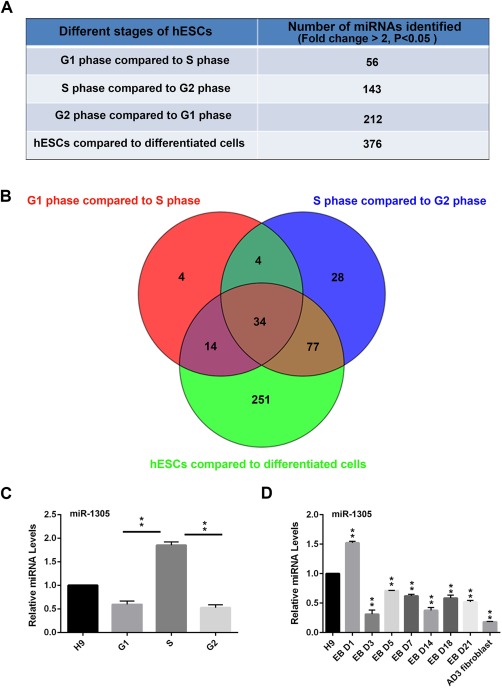
Identification of candidate miRNAs involved in maintenance of pluripotency and cell cycle regulation in pluripotent stem cells. **(A)**: Summary table displaying the number of candidate miRNA whose expression changed significantly (*p* < .05 and fold‐change > 2.0) during cell cycle stages or between hESCs and human placental fibroblasts; **(B)**: Venn diagram representation of candidate miRNAs shown in (A) and selection of 34 candidate miRNAs that were differently expressed between hESCs versus human placental fibroblast cells, S phase versus G1 phase, and S phase versus G2 phase; **(C)**: Quantitative RT‐PCR analysis of miR‐1305 expression in various cell cycle stages in hESC. Data is presented as mean ± SEM (*n* = 3). The unsynchronised control was set to 1.0 and all other values were calculated with respect to that. Significance was assessed using Student *t*‐test, ** *p* < .01; **(D)**: Quantitative RT‐PCR analysis of miR‐1305 expression during hESC differentiation using the Embryoid body method (EB). Ad3 fibroblasts – dermal skin fibroblasts from a 37‐year‐old Adult. Data is presented as mean ± SEM (*n* = 3). The H9 was set to 1.0 and all other values were calculated with respect to that. Significance was assessed using Student *t*‐test, ** *p* < .01.

The expression data generated from the array were normalized using the quintile normalization method 22 and differential miRNA expression method using the Limma package from Bioconductor 23. Statistical probability of significance (adjusted *p* values, set at *p* < .05) and a fold‐change > 2.0 were used to select the miRNAs that were differentially expressed between hESCs and human placental fibroblasts and those with significant expression changes between different stages of the hESCs cycle (Fig. 1A). A Venn diagram analysis was carried out (Fig. 1B) and 34 miRNAs were identified in the central shared area (Supporting Information Table 1). Recent studies have shown that the S and G2 phase of pluripotent stem cell cycle possess intrinsic properties toward the pluripotent state that are independent of G1 phase 24. For this reason, we focused our attention on one particular candidate, miR‐1305, which showed the highest expression in S phase of the cell cycle (Fig. 1C) alongside higher expression in hESCs when compared to human placental fibroblasts (Supporting Information Table 1), which suggest that this candidate may have important functions in regulating both cell cycle and hESCs differentiation.

To confirm the array expression studies we performed qRT‐PCR analysis. miR‐367, a well‐known miRNA that controls self‐renewal and pluripotency in hESCs and human induced pluripotent stem cells (hiPSCs), was used as a positive control 19. This indicated that the highest expression of miR‐1305 was observed during S phase of the cell cycle (Fig. 1C). Furthermore, qRT–PCR was used to test miR‐1305 expression during hESCs differentiation. Embryoid bodies (EB) were formed from hESCs and miR‐1305 expression analysed at time points throughout differentiation. Our results indicated that expression of miR‐1305 was upregulated at day 1 of hESCs differentiation followed by a significant downregulation from day 3 onward (Fig. 1D), thus corroborating the reduced expression in differentiated cells as shown from the array analysis. miR‐367 was highly expressed in G1 phase of the cell cycle (Supporting Information Fig. 1B) as well as significantly downregulated during hESCs differentiation (Supporting Information Fig. 1C) in agreement with previously published results 19.

### The Role of miR‐1305 in Regulating Pluripotency

**Figure 2:**
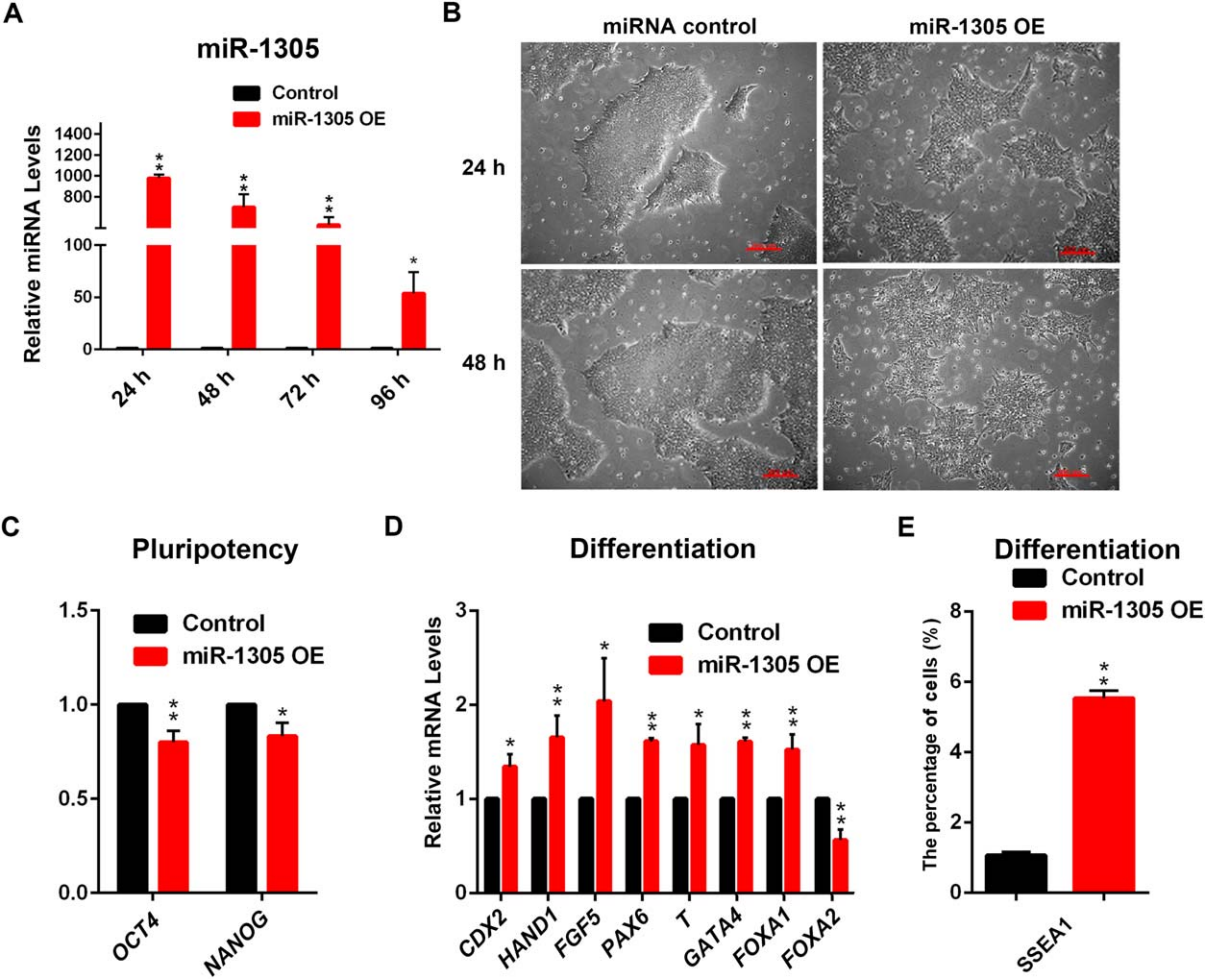
miR‐1305 overexpression induces hESCs differentiation. **(A)**: Quantitative RT‐PCR analysis of miR‐1305 expression after control miRNA overexpression or miR‐1305 overexpression (OE). Data is presented as mean ± SEM (*n* = 3). The miRNA overexpression control was set to 1.0 and all other values were calculated with respect to that. Significance was assessed using Student *t*‐test, *p <.05, **p <.01; **(B)**: Phase contrast observation of hESCs morphology at 24 and 48 hours post transfection with either control miRNA or miR‐1305 overexpression. Scale bars, 200 µm. This is a representative example of at least three independent experiments; **(C)**: Quantitative RT‐PCR analysis of the expression levels of pluripotency markers *OCT4* and *NANOG* in hESCs at 48 hours after transfection with control miRNA or miR‐1305 overexpression (OE). Data is presented as mean ± SEM (*n* = 3). The miRNA overexpression control was set to 1.0 and all other values were calculated with respect to that. Significance was assessed using Student *t*‐test, *p < .05, **p < .01; **(D)**: Quantitative RT‐PCR analysis of the expression levels of differentiation markers in hESCs at 48 hours after transfection with a miRNA overexpression control or miR‐1305 overexpression (OE). Data is presented as mean ± SEM (*n* = 3). The miRNA overexpression control was set to 1.0 and all other values were calculated with respect to that. Significance was assessed using Student *t*‐test, *p <.05, **p <.01; **(E)**: Flow cytometric analysis showing percentage of SSEA1 positive cells after overexpression of miR‐1305 at 48 hours after transfection. Data is presented as mean ± SEM (*n* = 3). Significance was assessed using Student *t*‐test, **p < .01.

To further investigate the function of miR‐1305 in hESCs, we overexpressed miR‐1305 using a miR‐1305 overexpression mimic (Thermo Fisher). Quantitative RT‐PCR analysis at different time intervals (24–96 hours) indicated that miR‐1305 expression peaked at 24 hours post‐transfection (>1000 fold) when compared to hESCs transfected with a scrambled miRNA overexpression control (Fig. 2A). We observed that cells transfected with the miRNA overexpression control maintained a typical hESCs morphology. At the same time, colonies in the miR‐1305 overexpression group were noticeably smaller and containing cells which had begun to lose the undifferentiated hESCs morphology, indicative of the onset of differentiation (Fig. 2B). Quantitative RT‐PCR analysis indicated a small but significant downregulation of the pluripotency markers *OCT4* and *NANOG* (Fig. 2C) and increased expression of primitive ectoderm (*FGF5*), neuroectoderm (*PAX6*), mesoderm (*T*), endoderm markers (*GATA4* and *FOXA1*), and trophectoderm markers (*CDX2* and *HAND1*; Fig. 2D). It is interesting to note that unlike other germ‐layer markers, the expression of *FOXA2*, a definitive endoderm marker, was downregulated upon miR‐1305 overexpression (Fig. 2D). Flow cytometric analysis also indicated an increase in the percentage of cells expressing the early differentiation marker, SSEA‐1 (Fig. 2E). In summary, the results from this analysis coupled with the early upregulation of miR‐1305 expression (from day 0 to day 1) suggest that miR‐1305 overexpression induces the early hESCs differentiation.

**Figure 3:**
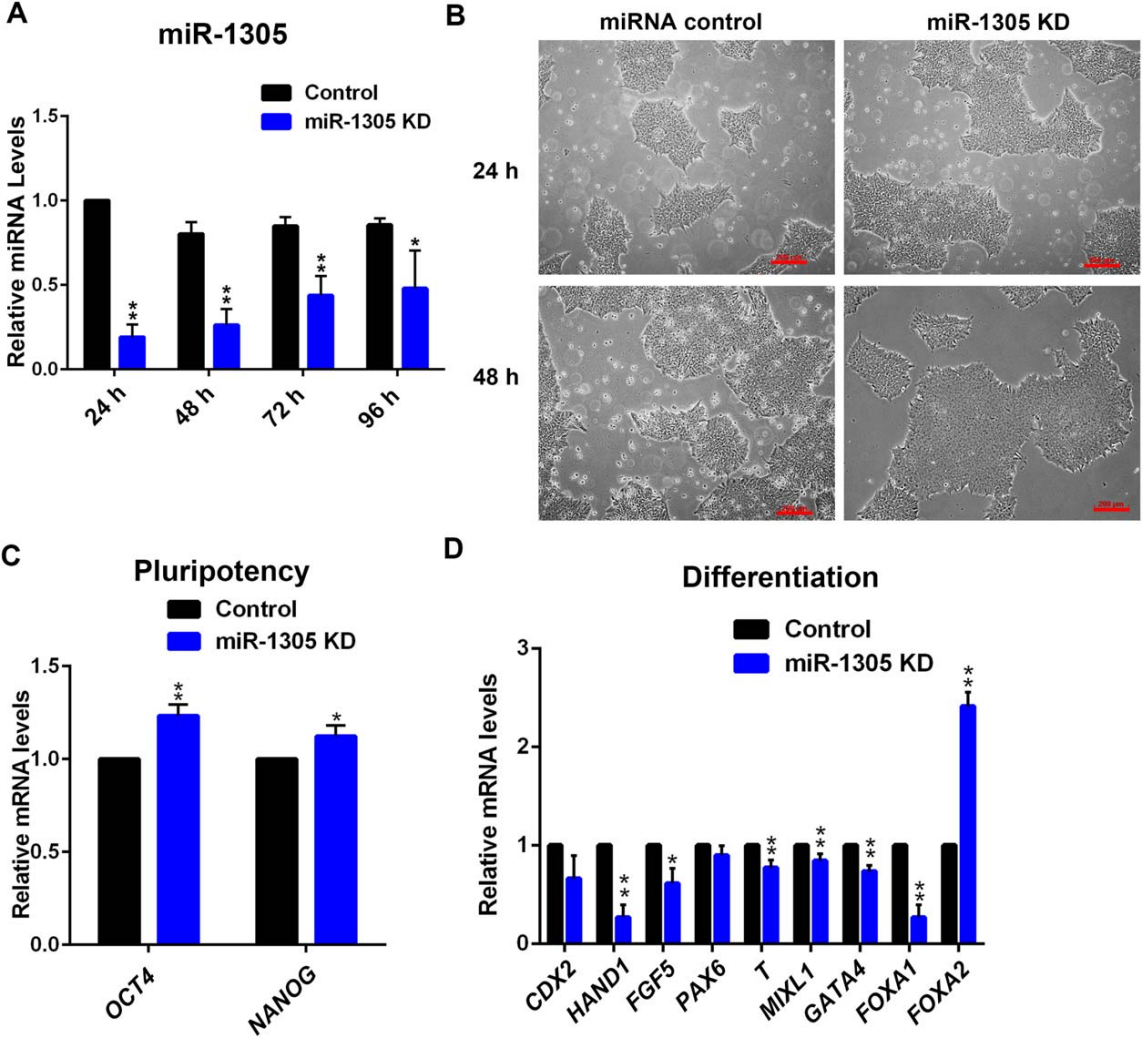
Knockdown of miR‐1305 in hESCs supports the maintenance of pluripotency. **(A)**: Quantitative RT‐PCR analysis of miR‐1305 expression after miRNA knockdown control or miR‐1305 knockdown transfection (KD). Data is presented as mean ± SEM (*n* = 3). The miRNA knockdown control was set to 1.0 and all other values were calculated with respect to that. Significance was assessed using Student *t*‐test, *p < .05, **p < .01; **(B)**: Phase contrast observation of hESCs morphology at 24 and 48 hours of transfection with miRNA knockdown control or miR‐1305 knockdown (KD). Scale bars, 200 µm. This is a representative example of at least three independent experiments; **(C)**: Quantitative RT‐PCR analysis of the expression levels of pluripotency markers *OCT4* and *NANOG* in hESCs at 48 hours after transfection with miRNA knockdown control or miR‐1305 knockdown (KD). Data is presented as mean ± SEM (*n* = 3). The miRNA knockdown control was set to 1.0 and all other values were calculated with respect to that. Significance was assessed using Student *t*‐test, *p < .05, **p < .01; **(D)**: Quantitative RT‐PCR analysis of the expression levels of differentiation markers in hESCs at 48 hours after transfection with miRNA knockdown control or miR‐1305 knockdown (KD). Data is presented as mean ± SEM (*n* = 3). The miRNA knockdown control was set to 1.0 and all other values were calculated with respect to that. Significance was assessed using Student *t*‐test, *p < .05, **p < .01.

To further understand the function of miR‐1305 in pluripotency, we performed loss of function studies by using miRNA‐inhibitors (Thermo Fisher). Quantitative RT‐PCR analysis indicated that the miR‐1305 inhibition significantly reduced miR‐1305 levels from 24–96 hours post transfection when compared to hESCs transfected with a scrambled miRNA control (Fig. 3A). Morphological assessment indicated that hESCs transfected with the control inhibitor or the miR‐1305 inhibitor, maintained their typical undifferentiated hESCs morphology (Fig. 3B), indicating that miR‐1305 inhibition did not induce hESCs differentiation. Furthermore, the colonies in the miR‐1305 inhibition group generally appeared to be bigger than in the control group and were devoid of the differentiated cells often observed at the edge of hESCs colonies (Fig. 3B). Quantitative RT‐PCR analysis indicated a slight but significant increase (10–20%) in the expression of the pluripotency markers *OCT4* and *NANOG* (Fig. 3C) and a reduction in the expression of primitive ectoderm (*FGF5*), mesoderm (*T* and *MIXL1*), endoderm (*GATA4* and *FOXA1*), and trophectoderm (*HAND1*) markers (Fig. 3D). It is interesting to note again, that unlike any other germ layer makers tested, the expression of *FOXA2*, a definitive endoderm marker, was significantly upregulated (2.5‐folds) upon miR‐1305 inhibition (Fig. 3D). These data suggest that miR‐1305 expression is likely to enhance the maintenance of hESCs pluripotency.

To investigate the role of miR‐1305 in hiPSC, we performed miRNA overexpression and inhibition transfection experiments in hiPSCs (Ad3 clone 1) generated from adult dermal skin fibroblasts and well characterised by our group. Quantitative RT‐PCR analysis indicated similar results to those from the hESC experiments: miR‐1305 overexpression reduced the expression of pluripotency markers *OCT4* and *NANOG* (data not shown) and increased the expression of ectoderm (*FGF5* and *PAX6*), mesoderm (*T*), endoderm (*GATA4* and *FOXA1*), and trophectoderm (*CDX2* and *HAND1*) markers (Supporting Information Fig. 2). When miR‐1305 was inhibited, we could detect an increase in the pluripotency markers *OCT4* and *NANOG* (data not shown) and a reduction in ectoderm (*FGF5* and *PAX6*), mesoderm (*T*), endoderm (*GATA4* and *FOXA1*), and trophectoderm (*CDX2* and *HAND1*) markers (Supporting Information Fig. 2). These results indicate that miR‐1305 functions are similar between hESCs and hiPSCs.

### The Role of miR‐1305 in Regulating Cell Cycle and Apoptosis

To test the function of miR‐1305 in cell cycle regulation, we synchronised hESCs at G2/M phase of the cell cycle by incubation with 200 ng/ml of the mitotic inhibitor nocodazole for 18 hours as described in Neganova et al. (2009) 20 and Zhang et al. (2009) 21. We assessed the percentage of cells in each stage of cell cycle by flow cytometric analysis as shown in Supporting Information Figure 3A. Upon release from G2/M accumulation, the hESCs cell cycle distribution was investigated by flow cytometry at regular intervals. This analysis indicated a higher percentage of cells in S phase and a lower percentage of cells in G1 phase of the cell cycle at 6 and 8 hours post‐release from G2/M accumulation when the miR‐1305 overexpressing group was compared to the control; however no changes were observed through G2 exit or entry (Supporting Information Fig. 3B). Inhibition of miR‐1305 expression resulted in increased cell accumulation in G1 phase at 10 hour time point. Furthermore a slightly higher percentage of cells was observed at G2 at this time point suggestive of a faster S to G2/M exit (Supporting Information Fig. 3C). The slower entry into S and faster exit from S to G2/M resulted in lower percentage of cells observed at S phase at this time point. Together these data suggest that overexpression of miR‐1305 speeds up the G1 to S transition, whilst its downregulation appears to slow down G1 to S transition and to slightly speed up the S to G2/M transition.

We noticed that overexpression of miR‐1305 results in formation of smaller hESCs colonies and presence of apoptotic cells in the culture media. This led us to investigate the impact of miR‐1305 overexpression and inhibition on pluripotent stem cell survival. Flow cytometric assays (Annexin V/PI and PARP activation) at 48 hours post transfection showed a higher percentage of apoptotic cells in the miR‐1305 overexpressing group compared to control (Supporting Information Fig. 4A). The opposite was observed upon downregulation of miR‐1305 (Supporting Information Fig. 4B). Together these data indicate that miR‐1305 plays an important role in cell cycle regulation and survival in pluripotent stem cells.

### Identification of *POLR3G* as a Direct Target of miRNA‐1305

To find potential miR‐1305 targets, we used several well‐known prediction softwares (TargetScan, miRDB, TargetMINER, RNA22‐HAS, and microRNA). To narrow down the candidate miR‐1305 targets, we focused on genes with known functions in the maintenance of pluripotency, cell cycle regulation or differentiation that were also predicted by more than one of the above mentioned softwares (Supporting Information Table 2). By comparing the software prediction results, three potential targets (*LIN28A*, *POLR3G*, and *ZIC2*) which have previously been implicated in maintenance of pluripotency in hESCs were chosen for further study (Supporting Information Table 3). *LIN28A* regulates the balance between stem cell differentiation and maintenance of pluripotency through targeting let‐7 25. *POLR3G,* a downstream target of *OCT4* and *NANOG,* is a novel pluripotency regulator in hESCs and is controlled by the ERK1/2 signalling pathway 26. *ZIC2* is a member of the ZIC family which is a negative regulator of Wnt signalling thought to be responsible for the formation of the brain signalling centre located at the midbrain‐hindbrain boundary in frog embryos 27.

**Figure 4:**
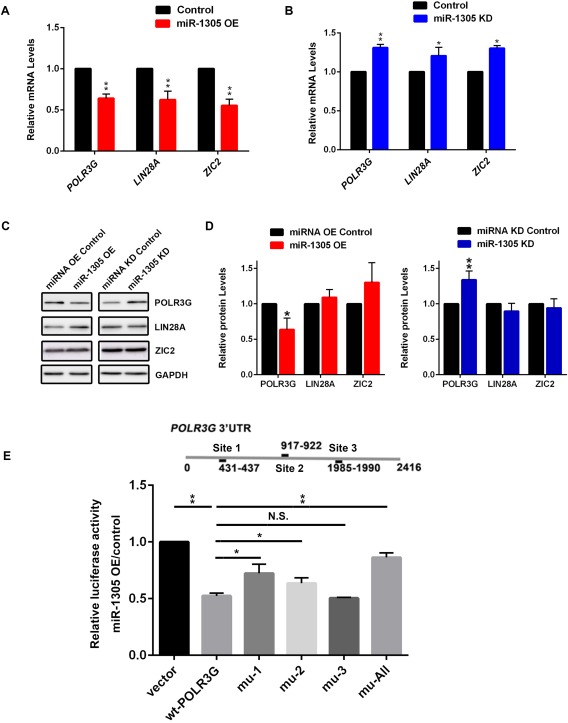
*POLR3G* is the downstream target of miR‐1305 in hESCs. **(A)**: Quantitative RT‐PCR analysis of the expression levels of the predicted miR‐1305 targets after miR‐1305 overexpression (OE). Data is presented as mean ± SEM (*n* = 3). The miRNA overexpression control was set to 1.0. Significance was assessed using Student *t*‐test, **p < .01; **(B)**: Quantitative RT‐PCR analysis of the expression levels of the predicted miR‐1305 targets after miR‐1305 knockdown (KD). Data is presented as mean ± SEM (*n* = 3). The miRNA knockdown control was set to 1.0 and all other values were calculated with respect to that. Significance was assessed using Student *t*‐test, *p < .05, **p < .01; **(C)**: Western blot analysis of the protein levels of POLR3G, ZIC2 and LIN28A. GAPDH served as a loading control. One representative example from three independent biological replicates is shown; **(D)**: Quantification of POLR3G, LIN28A and ZIC2 protein. The protein expression value of miRNA OE control or miRNA KD control was set as 1 and all the other values were calculated with respect to that. Data is presented as mean ± SEM (*n* = 3). Significance was assessed using Student *t*‐test, *p < .05, **p < .01. **(E)**: Dual luciferase reporter assays in hESCs co‐transfected with dual‐luciferase constructs containing wild‐type (wt‐*POLR3G*) or the *POLR3G* 3′‐UTR mutants (mu 1‐3 and mu‐All) together with the miRNA overexpression control or miR‐1305 overexpression mimic. The ratio between *Renilla* and Firefly luciferase activity was calculated. Then the ratio between transfections done on presence of miR‐1305 overexpression to miR overexpression control was calculated for each transfection resulting in luciferase activity value. The luciferase activity value of cells transfected with the empty luciferase vector was set as 1.0 and all the other values were calculated with respect to that. Data is presented as mean ± SEM (*n* = 3). Significance was assessed using Student *t*‐test, *p < .05, **p < .01, Abbreviation: N.S., not significant. Mu‐All includes mutations for all three identified miR‐1305 binding sites.

To confirm whether these three genes were bona fide targets, we performed quantitative RT‐PCR and Western Blot analysis. We characterized the mRNA expression levels of these predicted target genes in miR‐1305‐overexpressed and/or inhibited pluripotent stem cells using qRT‐PCR. Whilst the qRT‐PCR analysis showed the expected pattern of gene downregulation upon miR‐1305 overexpression and gene upregulation upon miR‐1305 inhibition (Fig. 4A and 4B), the Western Blot analysis confirmed this pattern only for POLR3G **(**Fig. 4C and 4D). From these data, we concluded that POLR3G is likely to be a direct downstream miR‐1305 target.

To confirm the direct regulation between miR‐1305 and *POLR3G*, we performed binding site predication for *POLR3G*. Three putative miR‐1305 binding sites were identified in the 3′UTR of *POLR3G* gene (Fig. 4E), all of which were conserved between different species (Supporting Information Table 3A). We investigated the functional interaction between miR‐1305 and the *POLR3G* 3′‐UTR by cloning the *POLR3G* 3′‐UTR into the psiCHECKTM‐2 vector (Promega) in order to create a reporter assay. This was used to investigate the binding specificity of miR‐1305 to *POLR3G* by mutating all three binding sites separately or together (Supporting Information Table 3B). The reporter assay indicated that the ectopic expression of miR‐1305 significantly suppressed (∼45%) the activity of the *POLR3G* reporter (Fig. 4E). Moreover, this inhibitory effect could be significantly abolished when binding site 1 (mu‐1), 2 (mu‐2) and all three sites (mu‐all) were mutated (Fig. 4E). Together, these data shows that miR‐1305 regulates *POLR3G* expression by binding to its 3′UTR.

### miR‐1305 Regulates the Pluripotency‐Differentiation Balance Through POLR3G

**Figure 5:**
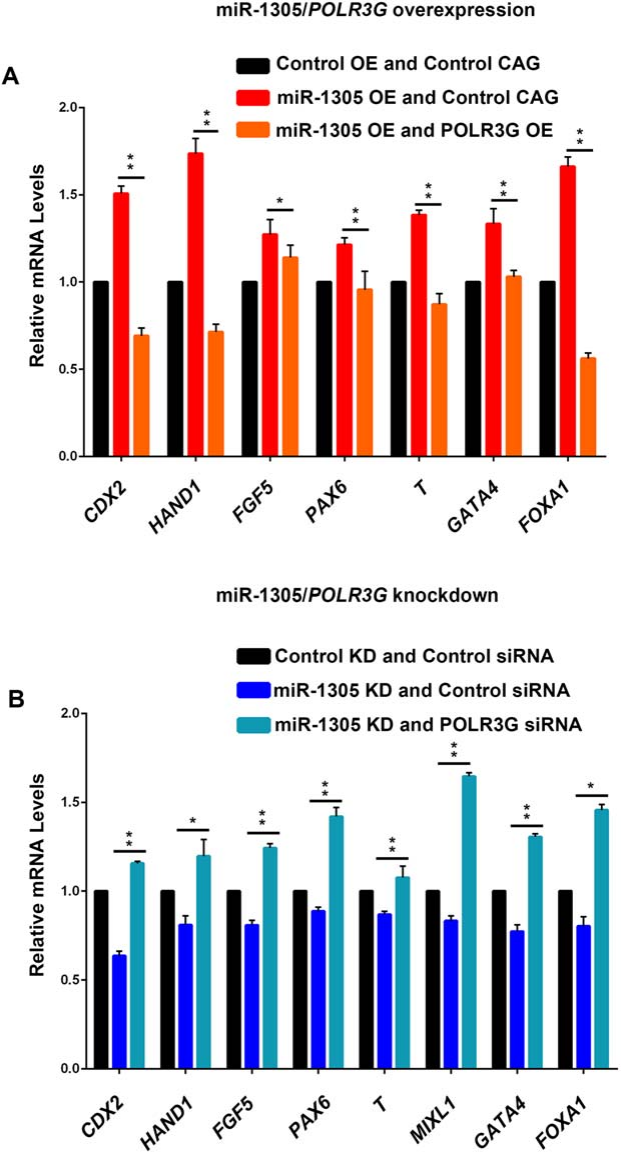
miR‐1305 regulates hESCs pluripotency‐differentiation balance through *POLR3G*. **(A)**: Quantitative RT‐PCR analysis of the expression levels of differentiation markers in the control‐CAG cell line or the POLR3G‐CAG cell line at the second day after transfection with a miRNA overexpression control or a miRNA‐1305 overexpression (OE). The miRNA overexpression control was set to 1.0 and all other values were calculated with respect to that. Data is presented as mean ± SEM (*n* = 3). Significance was assessed using Student *t*‐test, *p < .05, **p < .01; **(B)**: Quantitative RT‐PCR analyses of the expression levels of differentiation markers in hESCs at 48 hours after transfection with a miRNA knockdown control or miR‐1305 knockdown (KD) and control siRNA or *POLR3G* siRNA. The miRNA knockdown control was set to 1.0 and all other values were calculated with respect to that. Data is presented as mean ± SEM (*n* = 3). Significance was assessed using Student *t*‐test, *p < .05, **p < .01.

To test whether miR‐1305 regulates the balance between maintenance of pluripotency and onset of differentiation in pluripotent stem cells by targeting *POLR3G*, we utilised overexpression and knockdown of both *POLR3G* and miR‐1305 in hESCs. To test whether POLR3G overexpression could inhibit hESCs differentiation induced by miR‐1305 overexpression we generated a *POLR3G*‐overexpressing stable cell line. The coding region of *POLR3G* was cloned into a vector with a CAG‐promoter and a puromycin expression cassette and transfected into hESCs followed by puromycin selection (1.0 μg/ml). Both the mRNA (twofold higher) and the protein levels of POLR3G were increased in the stable cell line compared to the control (Supporting Information Fig. 5A and 5B). The POLR3G‐CAG line alongside an empty‐CAG control line was subjected to miR‐1305 overexpression in the following experimental groups: (1) miRNA overexpression control/control‐CAG, (2) miR‐1305 overexpression/control‐CAG, and (3) miRNA‐1305 overexpression/*POLR3G* overexpression. The relative levels of differentiated lineage markers were assessed by qRT‐PCR. As expected, miR‐1305 overexpression increased the levels of trophectoderm (*CDX2* and *HAND1*), primitive ectoderm (*FGF5*), neuroectoderm (*PAX6*), mesoderm (*T*), and endoderm (*GATA4* and *FOXA1*) markers (Fig. 5A). *POLR3G* overexpression abolished the increase of all these differentiation markers, indicating that miR‐1305 overexpression induced differentiation by regulating *POLR3G*.

To further confirm that miR‐1305 regulate hESC differentiation by targeting *POLR3G*, we utilised knockdown of *POLR3G* and inhibition of miR‐1305 in hESCs. The efficiency of knockdown was tested by qRT‐PCR. P*OLR3G* siRNA alone reduced *POLR3G* expression by 70% and miRNA/siRNA co‐inhibition reduced the *POLR3G* expression by 50% (Supporting Information Fig. 5C). The experiment was set up as three groups: (1) miRNA knockdown control/control siRNA; (2) miR‐1305 knockdown/control siRNA; (3) miR‐1305 knockdown/*POLR3G* siRNA and expression of markers of differentiation studied by qRT‐PCR. As expected, miR‐1305 inhibition significantly reduces (10–40%) the expression of all the differentiation markers, while this effect is abolished in the miR‐1305 inhibition and P*OLR3G* knockdown scenario where all the differentiation makers are upregulated compared to miR‐1305 inhibition alone (Fig. 5B). Together these data provide strong evidence that miR‐1305 impacts on the maintenance of hESCs pluripotency and the onset of their differentiation via binding directly to the 3′UTR of *POLR3G* and regulating its expression.

## discussion

Pluripotent stem cells hold great promise for basic biology studies, understanding of human development and as therapeutic options in cell based replacement therapies of inherited and age related degenerative diseases 28, 29, 30. However, to fully realise their potential and to avoid safety issues, directed and efficient strategies must be developed to control their differentiation along desired lineages. This requires a fundamental understanding of the molecular mechanisms underlying self‐renewal and maintenance of pluripotency. Along with signalling pathways, transcription factors and epigenetic regulators, miRNAs are emerging as important regulators in the maintenance of pluripotency and the cell cycle 16, 31. The interplay between cell cycle regulation and control of pluripotent stem cell differentiation represents a major interest for the stem cell field since differentiation to specific lineages has been shown to be highly dependent on the cell cycle stage. For example, hESCs in early G1 phase can only initiate differentiation into endoderm, whereas hESCs in late G1 are limited to neuroectoderm differentiation 10. In accordance with these published data, our group and others have shown that pluripotent stem cells residing in S and G2 are more effective at maintaining stemness 24, 32. Bearing this in mind, we set out to identify candidate miRNAs important for maintenance of pluripotency using two selection criteria: (1) significantly higher expression in S phase when compared to G1 and G2 phase of the cell cycle and (2) significantly higher expression in pluripotent stem cells when compared to somatic cells.

**Figure 6:**
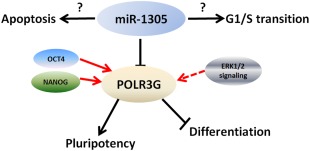
Schematic representation summarising the role of miR‐1305 in regulation of hESCs pluripotency‐differentiation balance, cell survival and cell cycle. Arrows indicate stimulatory modifications, blocked lines show inhibitory modifications and dashed lines indicate regulation mechanism that are still unclear, ? indicates scientific questions that have not yet been solved. Black lines represent data obtained from this manuscript, whereas red lines indicate data obtained from published literature.

We focused our attention on a novel miRNA, miR‐1305 and performed further functional studies to elucidate for the first time its role in pluripotent stem cells. These showed that expression of miR‐1305 is upregulated very rapidly upon the onset of differentiation (within 24 hours), indicating a role in early differentiation. Furthermore, miR‐1035 downregulation contributed to consolidation of pluripotent phenotype and its overexpression led to initiation of differentiation, thus suggesting that miR‐1305 acts as a regulator maintaining the fine balance between pluripotency and differentiation, presumably by modulating the expression of genes involved in this process. Using a combination of target prediction softwares and luciferase reporter assays, we identified *POLR3G* as a bona fide miR‐1305 target, thus providing for the first time evidence of a direct link between *POLR3G* and miR‐1305 in regulating hESC pluripotency. *POLR3G*, a downstream target of OCT4 and NANOG has been shown to play an important role in maintaining hESC pluripotency, as decreased levels of *POLR3G* result in loss of pluripotency and promote hESCs differentiation (Fig. 6), while its overexpression makes hESCs more resistant to differentiation 26. Our results fully corroborate these published findings and add another layer of molecular regulation by providing experimental evidence on regulation of pluripotency and differentiation through POLR3G/miR‐1305 mediated expression. Previous studies have shown that POLR3G is present in the cytoplasm of fertilized mouse zygotes and two‐cell embryos, but is located in the nucleus during the 8‐16‐cell and the blastocyst stage 26. Given our data on possible regulation of this gene by miR‐1305 in pluripotent stem cells, it would be of interest to investigate whether such interactions extend to the window proceeding epiblast development and which could be addressed using the naive pluripotent stem cell model.

It is of interest to note that *POLR3G* modulation impact hESCs in a more profound way than by modulating miR‐1305 expression. One possible explanation for this is that *POLR3G* inhibition and overexpression can be more easily achieved by directly targeting *POLR3G* rather than its regulator, miR‐1305. Another possibility is that miR‐1305 could be regulating other targets in addition to *POLR3G* and which may play different roles in pluripotency and differentiation. An example of this is *FOXA2* whose expression was downregulated upon miR‐1305 overexpression and vice versa. FOXA2 is a hepatocyte nuclear factor, shown to be important for transcriptional activation of liver‐specific genes 33. Downregulation of miR‐1305 at the pluripotent stem cells did not result in overexpression of other endodermal markers other than *FOXA2*, suggesting that a direct impact on differentiation to definitive endoderm through a FOXA2‐miR‐1305 interaction is unlikely at this early differentiation window. This could be due to the fact that similarly to embryonic development, delineation of key germ layers has not occurred as yet. However, it may be possible that a *FOXA2* overexpression mediated by miR‐1305 downregulation at the early stages of endodermal differentiation could provide a necessary and important cue for directing differentiation of human pluripotent stem cells to definitive endodermal lineages. This merits further investigation together with the possibility of miR‐1305 binding to *FOXA2* and regulating its expression.

Our study showed that miR‐1305 overexpression increased cells apoptosis while its knockdown reduced the population of apoptotic cells; however POLR3G has been shown to have no impact on pluripotent stem cell apoptosis 26, indicating that miR‐1305 is likely to regulate apoptosis through other targets. Indeed, target prediction software identified miR‐1305 binding sites in genes involved in regulation of apoptosis such as *BCL2, MDM2,* and *MDM4*. These results indicate that miR‐1305 might have potential functions in regulating apoptosis, but not via *POLR3G*. In addition to miR‐1305 involvement in apoptosis, our studies showed indicated that overexpression of miR‐1305 speeds up the G1/S entry. This together with the highest expression of this miRNA in S phase of the cell cycle led us to speculate that overexpression of miR‐1305 could be important for the maintenance of pluripotency. This was however contradicted by our miR‐1305 overexpression experiments which resulted in induction of differentiation in pluripotent stem cells and which suggest that miR‐1305 role in cell cycle regulation and maintenance of pluripotency could be achieved by regulation of different target genes. The target predictions software analysis identified *CDK6, CYCLIND2* and *RUNX2* as potential targets of miR‐1305, however only *RUNX2* showed the expected downregulation upon miR‐1305 overexpression and vice versa (data not shown), suggesting that the latter could be an additional target of miR‐1305. This possibility is further corroborated by a recent manuscript in which *RUNX2* was identified as one of the miR‐1305 targets 34. RUNX2 is an important osteogenic regulator shown to regulate the length of G1 phase of the cell cycle and apoptosis in highly proliferating mammary epithelial cells 35, 36. Given that miR‐1305 is also involved in cell cycle regulation and apoptosis in pluripotent stem cells, it would be of interest to further investigate whether these two processes are mediated via regulation of *RUNX2*.

To date very little is known about the function of miR‐1305. Three previous studies have shown a high expression of this miRNA in CpG island methylator‐phenotype positive or TP53‐mutated colon tumours 37, human periodontal ligament‐derived stem cells derived from smokers 34 and a lower expression in systemic lupus erythematosus and rheumatoid arthritis patient cells 38. Our manuscript is the first to describe a novel role for miR‐1305 in regulation of pluripotency‐early differentiation balance, cell cycle regulation and survival of pluripotent stem cells (Fig. 6). Whilst we have provided solid evidence that miR‐1305 in regulating pluripotency is mediated by targeting another pluripotency factor, *POLR3G*, further work is needed to understand how this single miRNA is also able to affect pluripotent self‐renewal, differentiation and survival.

## Materials and Methods

### Cell Culture

All hESCs and hiPSCs were cultured on Matrigel (growth‐factor‐reduced; BD Biosciences, NJ, USA) coated plates with mTeSR1 media (Stem Cell Technologies) according to Wicell Inc. protocols. Cells were passaged every 4–5 days at ∼80% confluence by using 0.02% EDTA (Versene, Waltham, MA, USA). All visible differentiated cells were manually removed before further passaging.

### Pluripotent Stem Cell Differentiation via the EB Method

The hESCs were cultured on Matrigel coated plate with mTeSR1 media. When 80%‐90% confluence was observed, the cells were digested by collagenase IV and resuspended in human EB media, KO‐DMEM (Thermo Fisher, Waltham, MA, USA), 1% l‐glutamine (Thermo Fisher, Waltham, MA, USA), 1% nonessential amino acids (Thermo Fisher, Waltham, MA, USA), 20% Fetal Bovine Serum (Thermo Fisher, Waltham, MA, USA). Three wells of hESCs colonies were transferred to one well of the ultra‐low attachment six‐well plates (Corning, New York, USA) for EB formation.

### Cell Cycle Synchronization

For cell cycle synchronization, the hESCs were collected, processed as previously described by Neganova *et al*. 20 and Zhang et al. 21.

### Cell Cycle Analysis

Cell cycle analysis was performed using Cycletest Plus DNA Reagent Kit (BD, NJ, USA). hESCs were harvested by accutase treatment and counted with a hemocytometer. 500,000 cells were fixed, permeabilized, and stained in accordance with the manufacturers' instructions, and the sample was analyzed by flow cytometry using a FACSCalibur measuring FL2 area versus total counts. The data were analyzed using ModFit (Tree Star, Inc., http://mycyte.org/) and FlowJo (Tree Star, Inc., http://mycyte.org/) softwares to generate the percentages of cells in G1, S, and G2 to M phases of the cell cycle.

### Apoptosis Assay

Apoptosis was detected using the Annexin‐V‐FITC apoptosis detection kit and 647 Mouse anti‐Cleaved PARP (BD Bioscience, NJ, USA). The protocol was performed in accordance with the manufacturer's instructions. The hESCs were collected processed and analysed as previously described (Zhang *et al*. 2009) 21.

### miRNA Expression Analysis

Agilent human miRNA (V3) 8X15K microarray were used for miRNA analysis of each sample with three biological replicates. Total RNA was extracted from unsynchronised and synchronised hESCs using the *mir*Vana miRNA Isolation Kit (Ambion, Waltham, MA, USA) according to the manufacturer's instructions. RNA quality was assessed using the RNA 6000 Nano Kit on the 2100 Bioanalyzer instrument (Agilent Technologies, Santa Clara, CA, USA) according to the manufacturer's instructions.

### RNA Extraction, Reverse Transcription and qRT‐PCR

Total RNA isolation was performed using the ReliaPrep RNA Cell Minprep System (Promega). RNA quality was evaluated using the NanoDrop 2000 spectrophotometer (Thermo Fisher). Total RNA was reverse‐transcribed into cDNA using GoScript Reverse Transcription System. qRT‐PCR was performed using the QuantStudio 7 Flex Real‐Time PCR System (Life Technologies, Waltham, MA, USA), and the GoTaq qPCR Master (Promega, Madison, WI, USA) according to the manufacturer's instructions. Primer sequences used for qRT‐PCR are provided in Supporting Information Table 4.

### miRNA Reverse Transcription and qRT‐PCR

TaqMan MicroRNA Reverse Transcription Kit was used for miRNA reverse transcription. One microgram of total RNA sample were used per miRNA RT reaction. TaqMan MicroRNA probe (Thermo Fisher) was used as the miRNA Reverse Transcription (RT) primer. qRT‐PCR was performed using the QuantStudio 7 Flex Real‐Time PCR System (Thermo Fisher, Waltham, MA, USA), and the TaqMan Small RNA Assays kit (Thermo Fisher, Waltham, MA, USA) according to the manufacturer's instructions.

### Western Blotting

Protein (30 µg) from whole cell extracts was used for western blotting analysis. Antibodies used for Western Blotting are as follows: ZIC2 (1:1000, Abcam, Cambridge, MA, USA), LIN28A (1:1000, Santa Cruz Biotechnology, Inc., Santa Cruz, CA, USA), GAPDH (1:750, Abcam), POLR3G (1:500, Santa Cruz Biotechnology, Santa Cruz, CA, USA)

### Small Interfering RNAs and miRNA Transient Transfection

*POLR3G* siRNAs were obtained from Thermo Fisher (4390824). The hESCs were transfected with si‐control or si‐POLR3G by Lipofectamine RNAiMAX Transfection Reagent according to the manufacturer's instructions.miRNA mimics are small, chemically modified double‐stranded RNAs and miRNA inhibitor are small, chemically modified single‐stranded RNAs. The hESCs and hiPSCs were transfected with 60 pmoles miRNA mimic (Invitrogen Cat. #4464066) or inhibitor (Invitrogen Cat. #4464084) by Lipofectamine RNAiMAX Transfection Reagent according to the manufacturer's instructions. In brief, the hESCs and hiPSCs were first dissociated by incubating with EDTA (0.02%). 3 × 10^5^ cells were seeded into one well of a 12 well plate one day before lipofection. 6μl Lipofectamine® RNAiMax reagent and 60 pmoles miRNA mimic or inhibitor were used for transfection of one well.

### Generation of a *POLR3G* Overexpressing Stable Cell Line

The *POLR3G* full coding sequences was isolated from cDNA generated from hESC line using the following oligonucleotides: *POLR3G* Forward 5′‐GCG ATC GCA AVA TGG ATG AGG C‐3′ and *POLR3G* Reverse 5′‐ GCG GCC GCT TGT TTG TGG TGA TTG‐3′. The full length fragment was ligated into the pCAG‐IP vector. hESCs were transfected with control empty Vector or *POLR3G* construct by Lipofectamine 3000 (Invitrogen, Waltham, MA, USA) following manufacturer's instructions. 2 days after transfection, stable clones were selected by using puromycin (1.0 µg/ml) for 7 days. The positive clones were expanded in mTeSR1 with 0.5 µg/ml puromycin. The expression level of POLR3G in stable cell lines was assessed by qRT‐PCR and western blotting.

### Luciferase Assays

The cells were seeded at a density of 1 × 10^5^ per well into 24‐well plates 24 hours before transfection. To examine the effect of miR‐1305 on the *POLR3G* reporter, control vector or *POLR3G* expression plasmids, miRNA overexpression control or miR‐1305 overexpression were co‐transfected with the reporter by Lipofectamine 3000 (Thermo Fisher, Waltham, MA, USA) following manufacturer's instructions. Cell extracts were prepared 48 hours after transfection. Luciferase activities were evaluated with a Dual‐Luciferase Assay System (Promega, Madison, WI, USA) according to the manufacturer's recommendations.

### Statistical Analysis

Quantitative data are expressed as means ± SEM, *n* = 3. Statistical significance was determined by the Student's t‐test. *p*‐value <.05 was considered as statistically significant (*p <.05, **p <.01).

## Conclusion

In summary, our results suggest that miR‐1305 regulate pluripotency, cell survival and cell cycle in human Pluripotent Stem cells. Overexpression of miR‐1305 induced differentiation of pluripotent stem cells, increased cell apoptosis and sped up G1/S transition, while its downregulation facilitated the maintenance of pluripotency and increased cell survival. miR‐1305 regulate the balance between maintenance of pluripotency and differentiation by targeting POLR3G. Overexpression of POLR3G rescued pluripotent stem cell differentiation induced by miR‐1305 overexpression. In contrast, knock‐down of POLR3G expression abolished the miR‐1305‐knockdown mediated enhancement of pluripotency, thus validating its role as miR‐1305 target in human pluripotent stem cells.

## Author Contributions

S.J. performed research, data analysis, figure preparation, manuscript writing, and final approval of manuscript; J.C. performed some of the research, data analysis, and final approval of manuscript; L.Z. performed some of the research and final approval of manuscript; D.M. performed data analysis and final approval of manuscript; L.A. contributed to manuscript writing, final approval of manuscript, and fund raising; I.N. performed data analysis, manuscript writing, figure preparation, design of the study, and final approval of manuscript; M.L. designed research, data analysis, figure preparation, manuscript writing, fund raising, and final approval of manuscript.

## Potential Conflicts of Interest

All authors declared no potential conflicts of interest.

## Supporting information

Supplementary Information Figure 1

Supplementary Information Figure 2

Supplementary Information Figure 3

Supplementary Information Figure 4

Supplementary Information Figure 5

Supplementary Information Table 1

Supplementary Information Table 2

Supplementary Information Table 3

Supplementary Information Table 4
