# Supplementary figures and images for: A Novel Role for miR‐1305 in Regulation of Pluripotency‐Differentiation Balance, Cell Cycle, and Apoptosis in Human Pluripotent Stem Cells
Source: Stem Cells. 2016 Jul 11;34(9):2306–17. doi: 10.1002/stem.2444 (PMC5031214; doi:10.1002/stem.2444)

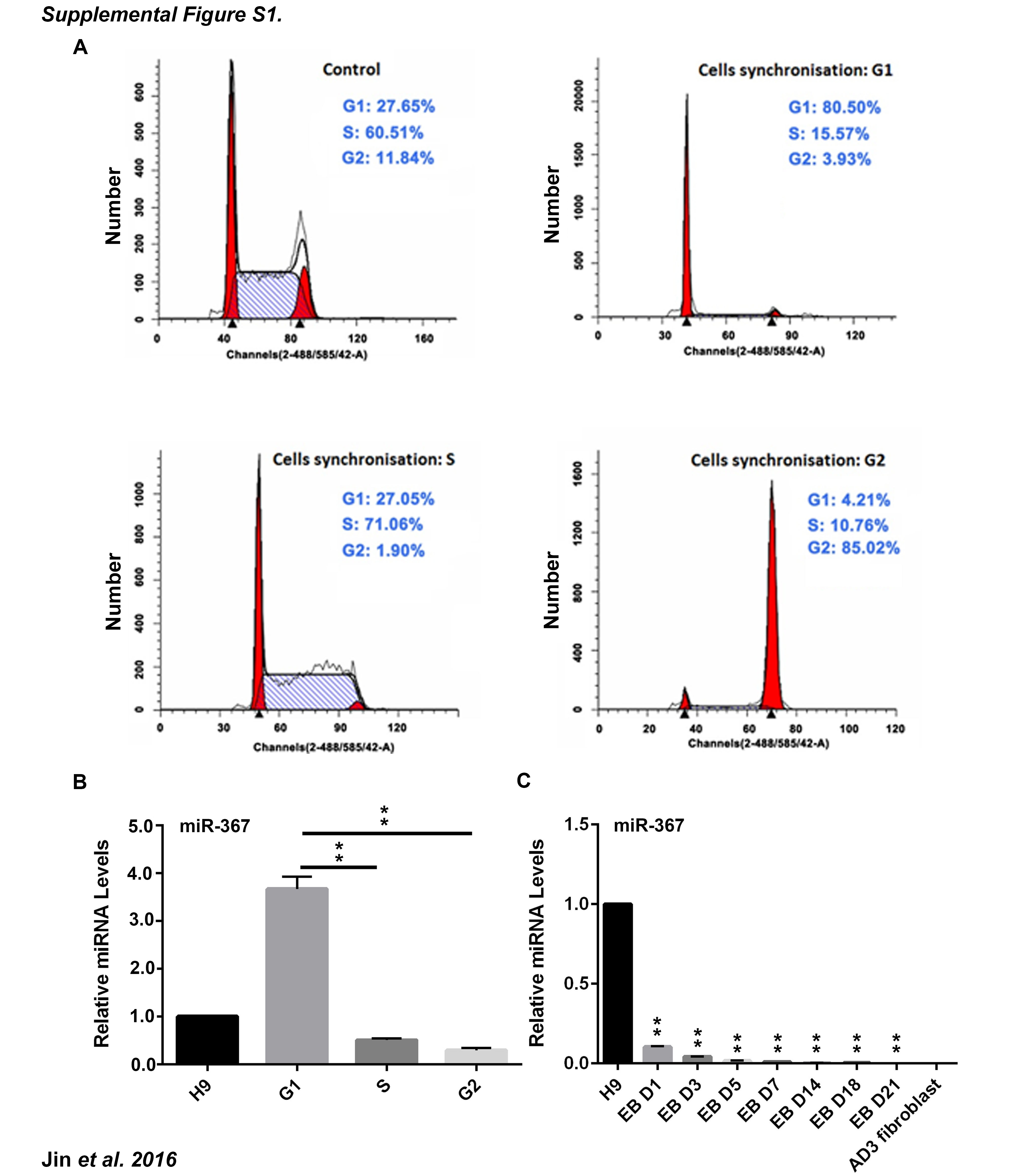

Supplement: Supplementary file 1 — Supplementary Information Figure 1 [file STEM-34-2306-s001.tif]

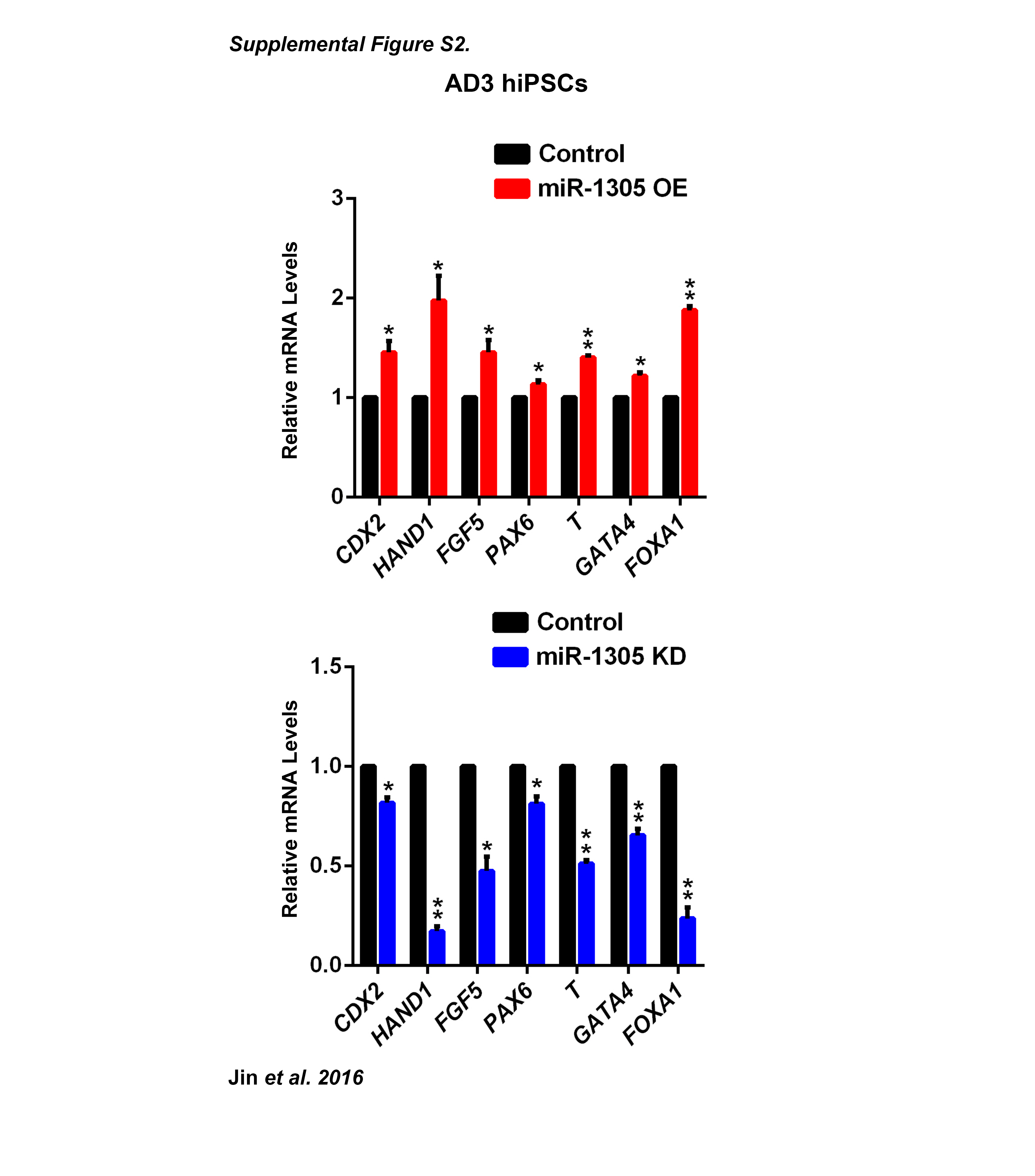

Supplement: Supplementary file 2 — Supplementary Information Figure 2 [file STEM-34-2306-s002.tif]

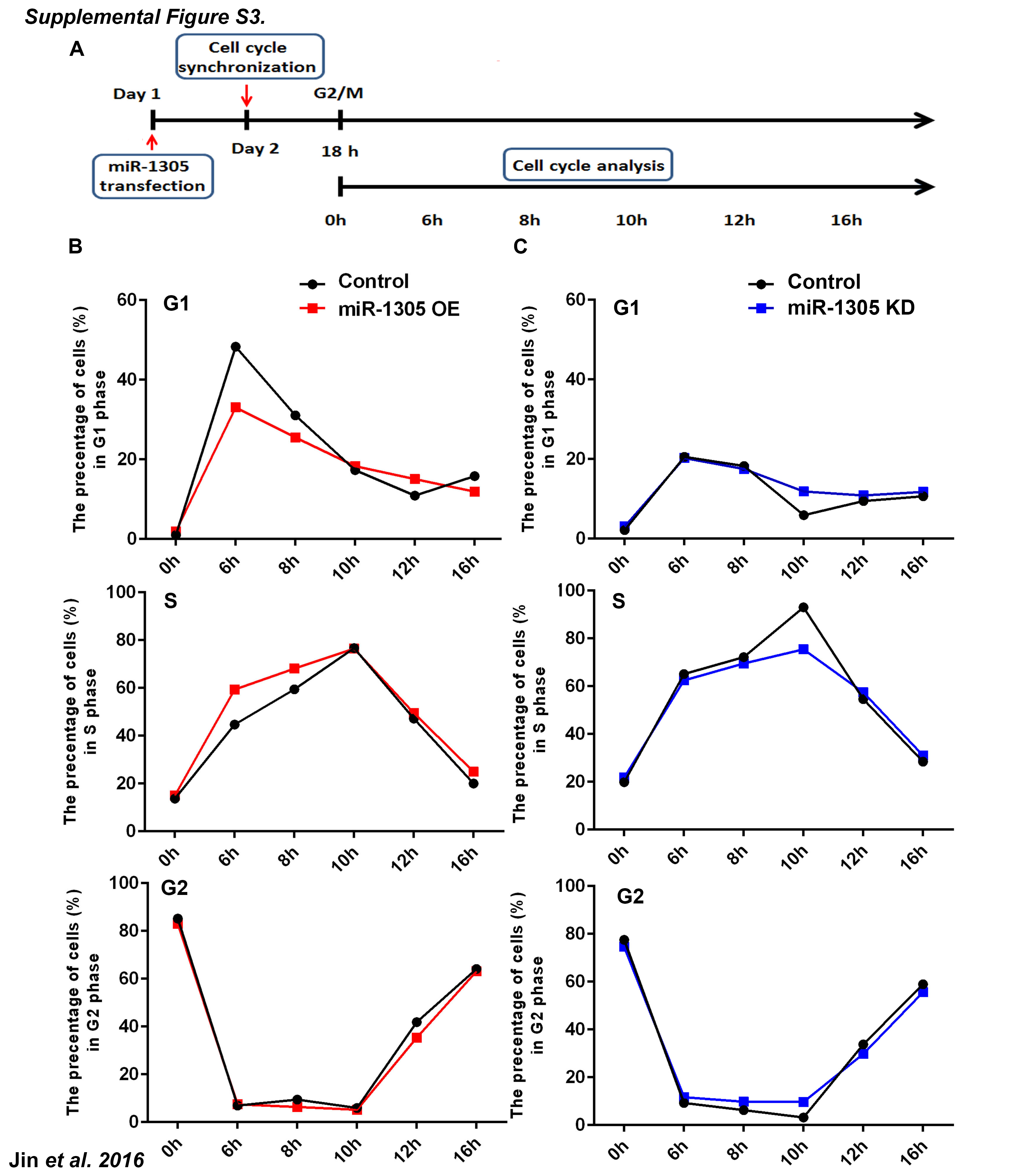

Supplement: Supplementary file 3 — Supplementary Information Figure 3 [file STEM-34-2306-s003.tif]

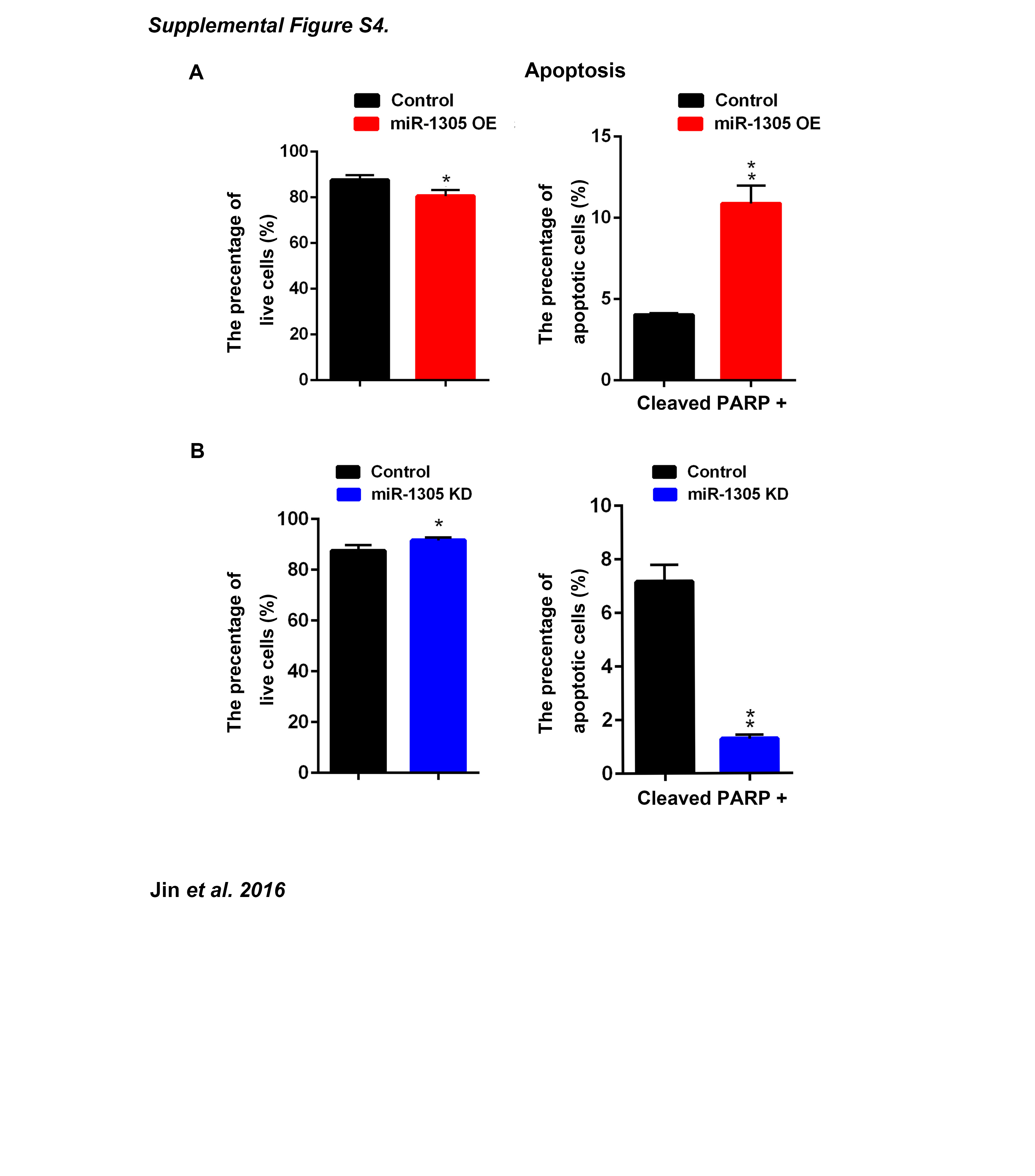

Supplement: Supplementary file 4 — Supplementary Information Figure 4 [file STEM-34-2306-s004.tif]

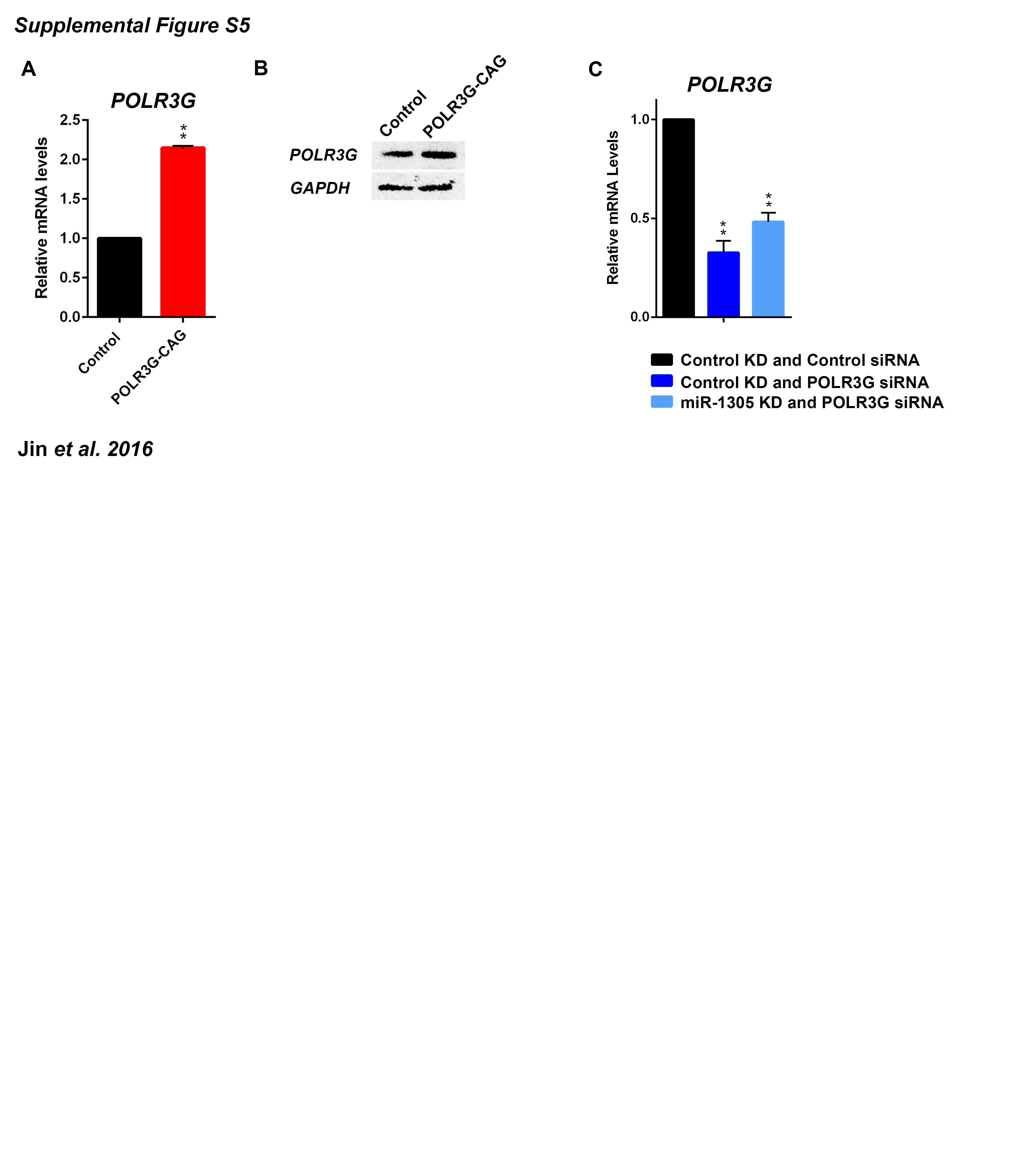

Supplement: Supplementary file 5 — Supplementary Information Figure 5 [file STEM-34-2306-s005.tif]

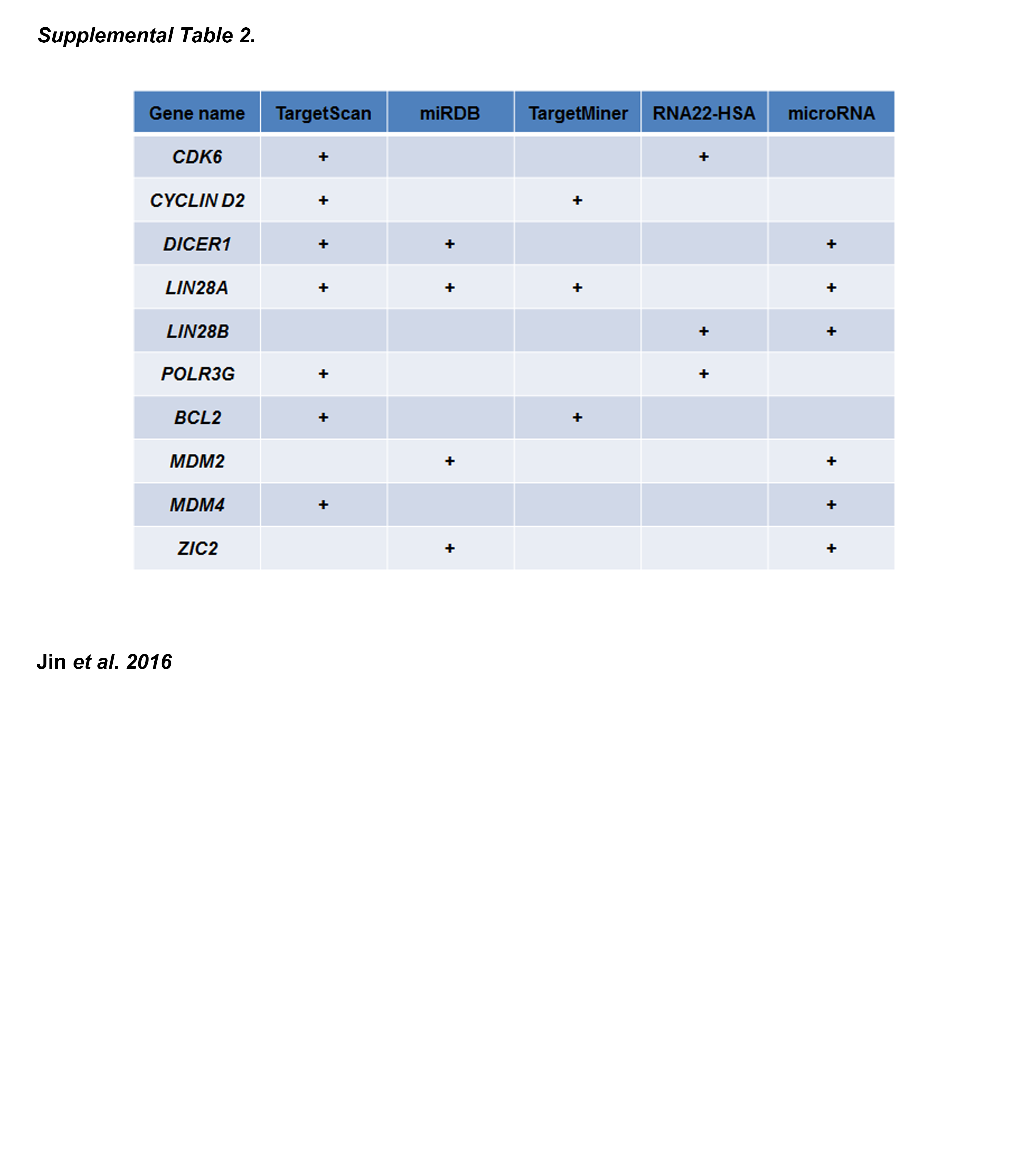

Supplement: Supplementary file 7 — Supplementary Information Table 2 [file STEM-34-2306-s007.tif]

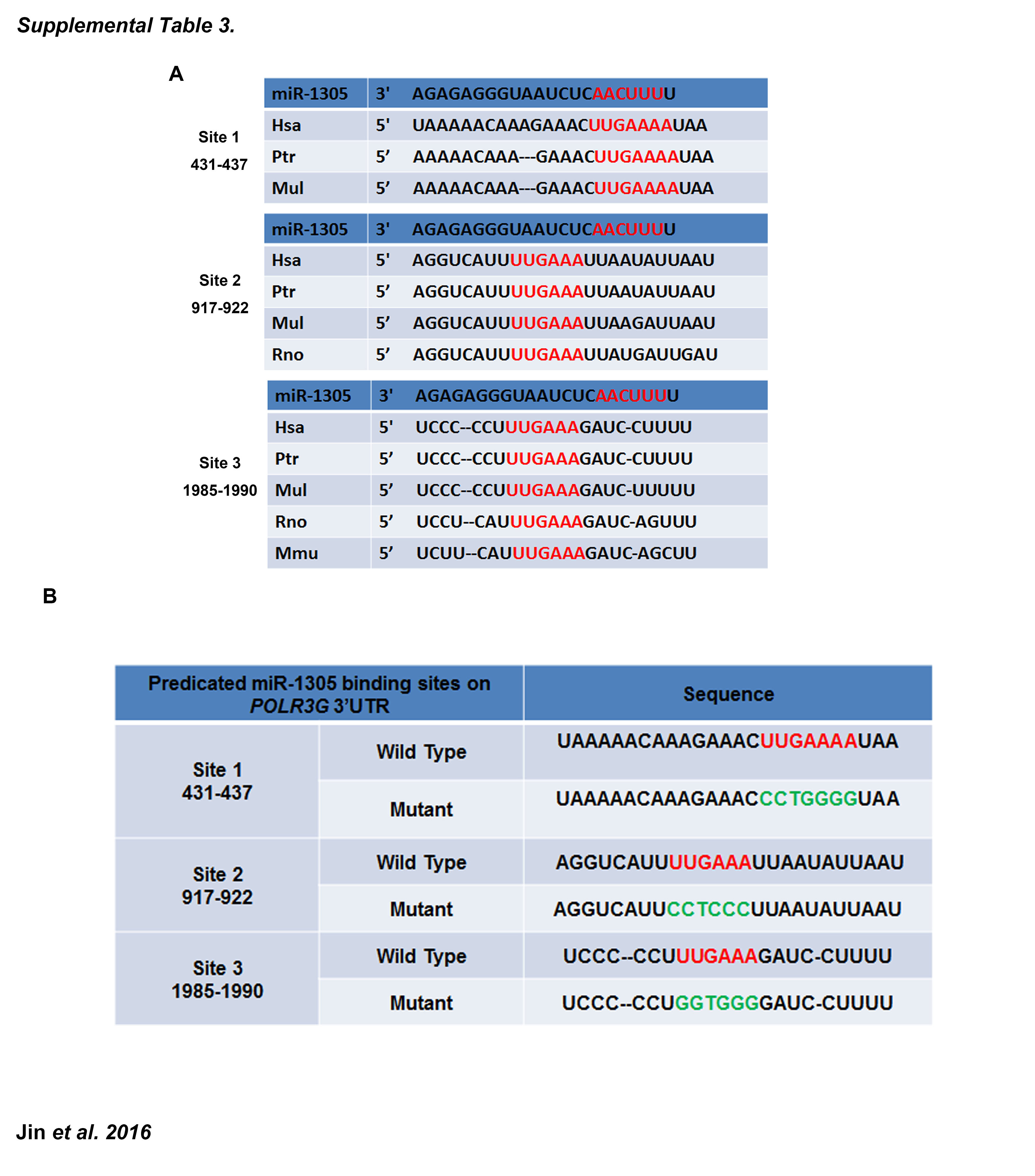

Supplement: Supplementary file 8 — Supplementary Information Table 3 [file STEM-34-2306-s008.tif]
